# Evoked Potentials and EEG Analysis in Rett Syndrome and Related Developmental Encephalopathies: Towards a Biomarker for Translational Research

**DOI:** 10.3389/fnint.2020.00030

**Published:** 2020-05-28

**Authors:** Joni N. Saby, Sarika U. Peters, Timothy P. L. Roberts, Charles A. Nelson, Eric D. Marsh

**Affiliations:** ^1^Lurie Family Foundations MEG Imaging Center, Department of Radiology, The Children’s Hospital of Philadelphia, Philadelphia, PA, United States; ^2^Department of Pediatrics, Vanderbilt University Medical Center, Nashville, TN, United States; ^3^Department of Radiology, Perelman School of Medicine at the University of Pennsylvania, Philadelphia, PA, United States; ^4^Laboratories of Cognitive Neuroscience, Division of Developmental Medicine, Boston Children’s Hospital, Harvard Medical School, Boston, MA, United States; ^5^Division of Neurology and Pediatrics, Children’s Hospital of Philadelphia, Philadelphia, PA, United States; ^6^Departments of Neurology and Pediatrics, Perelman School of Medicine at the University of Pennsylvania, Philadelphia, PA, United States

**Keywords:** biomarker, Rett syndrome, developmental encephalopathy, evoked potential, EEG

## Abstract

Rett syndrome is a debilitating neurodevelopmental disorder for which no disease-modifying treatment is available. Fortunately, advances in our understanding of the genetics and pathophysiology of Rett syndrome has led to the development of promising new therapeutics for the condition. Several of these therapeutics are currently being tested in clinical trials with others likely to progress to clinical trials in the coming years. The failure of recent clinical trials for Rett syndrome and other neurodevelopmental disorders has highlighted the need for electrophysiological or other objective biological markers of treatment response to support the success of clinical trials moving forward. The purpose of this review is to describe the existing studies of electroencephalography (EEG) and evoked potentials (EPs) in Rett syndrome and discuss the open questions that must be addressed before the field can adopt these measures as surrogate endpoints in clinical trials. In addition to summarizing the human work on Rett syndrome, we also describe relevant studies with animal models and the limited research that has been carried out on Rett-related disorders, particularly methyl-CpG binding protein 2 (MECP2) duplication syndrome, CDKL5 deficiency disorder, and FOXG1 disorder.

## Introduction

Rett syndrome is a genetic neurodevelopmental disorder that affects predominantly females. Estimated to occur in 1 of every 10,000 female births, Rett syndrome is characterized by near-normal growth and development for the first 6–18 months of life followed by a deceleration of development and loss of previously acquired skills, including spoken language and purposeful hand use (Hagberg, 1985; Neul et al., 2010). Other symptoms include stereotypic hand movements, gait apraxia, seizures, breathing abnormalities, sleep disturbances, and scoliosis, although the presence and severity of these features vary from person to person. In over 95% of cases, Rett syndrome is caused by mutations in the X-linked methyl-CpG binding protein 2 (MECP2) gene (Amir et al., 1999; Neul et al., 2014). Disease severity is largely dependent on the type of MECP2 mutation (Bebbington et al., 2008; Neul et al., 2008; Cuddapah et al., 2014), although two individuals with the same mutation can appear significantly different due to other contributing factors including genetic background and patterns of X-chromosome inactivation.

Treatment options for Rett syndrome are currently very limited. However, over the past several decades, significant progress has been made in understanding the genetic, cellular, and molecular mechanisms of the disorder (Leonard et al., 2017; Ip et al., 2018; Vashi and Justice, 2019). Advances in the understanding of the underlying pathophysiology have led to the development of new therapies, namely symptomatic pharmacologic interventions that act on the downstream cellular pathways affected in Rett syndrome, as well as gene therapy approaches that target the MECP2 gene directly. The effectiveness of these treatments in animal models of Rett syndrome has created enthusiasm within the Rett community as well as hope for a cure for the condition (van Karnebeek et al., 2016; Clarke and Abdala Sheikh, 2018). However, despite the efficacy of these treatments at the preclinical level all of the treatments that have preceded to clinical trials have so far failed to show the anticipated effects (Glaze et al., 2009, 2017; Khwaja et al., 2014; O’Leary et al., 2018).

The recurrence of failed clinical trials is not unique to Rett syndrome and has also been a point of concern for other neurodevelopmental disorders including Fragile X syndrome (Berry-Kravis et al., 2016; Erickson et al., 2017) and autism spectrum disorder (King et al., 2009; Aman et al., 2017; Veenstra-VanderWeele et al., 2017). Although a variety of factors may have contributed to the failure of these trials, one likely factor concerns the lack of sensitivity of the selected outcome measures (Jeste and Geschwind, 2016; Sahin et al., 2018). Indeed, the primary outcome measures for most extant clinical trials for neurodevelopmental disorders have been a caregiver and/or clinician impression of the child’s symptoms, which are subject to placebo effects and may obscure small improvements that do not manifest clinically. Given the issue of failed trials in Rett syndrome and other neurodevelopmental disorders, it has become increasingly clear that there is an immense need for objective biological markers of central nervous system function to improve the prospects of novel therapeutics. Ideally, biomarkers or other quantitative measures would replace caregiver/clinician reports as the primary efficacy endpoints of clinical trials to provide a more sensitive measure while mitigating the subjectivity of parent or caregiver reports and may shed light on underlying neural mechanisms (Levin and Nelson, 2015).

Biomarkers of central nervous system function are typically derived from either functional magnetic resonance (MR) imaging (fMRI) or electrophysiological [electroencephalography (EEG) or magnetoencephalography] modalities. Due to the restricted nature of the MR environment and the necessity for the subject to remain still, acquiring fMRI data from participants with Rett syndrome would require sedation, which introduces a range of medical risks and precludes the possibility of examining higher-order sensory and/or cognitive processes. EEG on the other hand, is notably less constraining and allows some movement on part of the participant. Therefore, EEG can be used with individuals with Rett syndrome without requiring sedation, and thus represents a key advantage over fMRI for measuring brain activity in this population. Another fundamental benefit of EEG is its scalability due to its low cost, wide availability, relative ease of use.

EEG measures typically focus on quantifying neural responses to a repeated sensory stimulus (evoked potentials, EPs) or characterizing on-going background activity during rest or sleep (resting state). EPs can be elicited using the passive presentation of a sensory (auditory, visual, or somatosensory) stimulus, without requiring overt effort or a behavioral response on part of the participant. Similarly, resting-state EEG can be acquired from a subject while their attention is diverted by another activity such as bubbles or a silent movie. Therefore, both of these approaches can work with severely impaired populations, such as individuals with Rett syndrome.

This review aims to summarize the existing EP and EEG studies of Rett syndrome and describe how we can build on this work to begin applying EP and EEG measures as surrogate endpoints in clinical trials. We will also describe relevant EEG studies that have been conducted for related developmental encephalopathies (DEs), specifically MECP2 duplication syndrome, CDKL5 deficiency disorder (CDD), and FOXG1 disorder. Similar to children with Rett syndrome, children with MECP2 duplication syndrome, CDD, and FOXG1 disorder exhibit intellectual impairment, breathing abnormalities, apraxia, and epilepsy with a progressive postnatal onset (Paciorkowski et al., 2018). Given the overlap in symptomatology, many individuals with these disorders were frequently considered variants of Rett syndrome. However, ongoing clinical research has revealed that in addition to having unique genetic etiologies, each of these disorders has a unique set of symptoms and a characteristic clinical course that distinguish them from Rett syndrome and one another (Fehr et al., 2013; Lim et al., 2017; Paciorkowski et al., 2018). Very few EP or EEG studies have focused on MECP2 duplication syndrome, CDD, or FOXG1 disorder. This omission is likely due in part to the fact that these other disorders have only recently been recognized and the number of affected children (thus the number of potential research participants) is notably more restricted. Therefore, the present review will concentrate on Rett syndrome, although we describe findings from the other disorders when available.

In addition to describing the extant human research, we also summarize the relevant preclinical work with animal models of Rett syndrome, MECP2 duplication syndrome, CDD, and FOXG1 disorder to highlight the shared and disparate aspects of the preclinical models which are used for treatment development. We conclude with suggestions for future research, including how increased coordination between preclinical and human studies will further facilitate the identification of reliable biomarkers and ultimately, the development of effective treatments.

## Review of Existing Studies

### Auditory Evoked Potentials

Concerning EPs in Rett syndrome, the most thoroughly studied sensory domain has been the auditory system. Many of the early studies in this area focused exclusively on auditory brainstem responses (ABRs). The results of these studies were inconsistent, with several studies reporting normal ABRs in participants with Rett syndrome (Verma et al., 1987; Kálmánchey, 1990; Stach et al., 1994) and others reporting differences between Rett and typically developing (TD) groups (Bader et al., 1987, 1989b; Pelson and Budden, 1987; Pillion et al., 2000, 2010). The inconsistency in findings may be attributed in part to the use of small sample sizes and variability in the ages and clinical profiles of the individuals tested. Methodological differences, including the selected comparison group and use of sedation in some studies (Pelson and Budden, 1987; Pillion et al., 2000) and not others (Stach et al., 1994), may have also contributed to the mixed results (Pillion et al., 2010). When group differences were observed, they were mostly in the latency of the later aspects of the ABR, specifically wave V and the wave III-V complex, with normal values for the earlier components.

**Figure 1:**
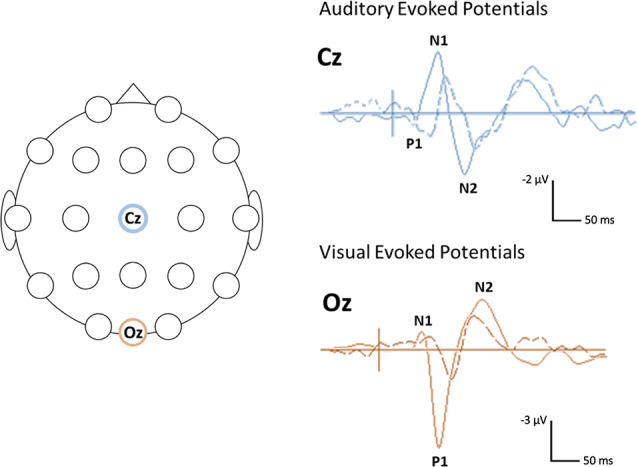
Example of visual and auditory evoked potential (AEP) waveforms and head locations. Sample (unpublished) auditory and visual evoked potentials (AEPs/VEPs) from a 16-year-old individual with Rett syndrome (dotted line) and an age-matched typically developing (TD) control (solid line) The primary positive (P) and negative (N) components are indicated. The schematic on the left shows the location of the electrodes used for the auditory (Cz) and visual (Oz) responses. The auditory response was elicited using a 500 Hz tone. The visual response was elicited using a reversing checkerboard. Negativity is plotted up.

In contrast to the mixed findings on ABRs, studies that have considered the subsequent (middle and cortical) components of the auditory evoked potential (AEP) have consistently noted atypical responses in Rett syndrome, at least in a subset of participants (see Table 1 for a summary of studies; Bader et al., 1989b; Stach et al., 1994; Stauder et al., 2006; Foxe et al., 2016). Those studies that have examined middle latency responses (MLR) have found the Pa component of the MLR to be absent or delayed in about half of the participants tested (Bader et al., 1989b; Stach et al., 1994). Both of these studies also reported atypical cortical responses at the vertex electrode, with Stach et al. reporting a complete absence of the N1 and P2 components in many participants. Bader et al., 1989b were able to identify N1 and P2 components in all of the participants enrolled in their study, although the latencies of these components were substantially delayed in several participants and as a group overall. More recent work by Foxe et al. has provided further evidence for atypical AEPs in Rett syndrome. In this study, gross differences were observed in both the timing and morphology of the late cortical response, including marked attenuation of the N1—P2 complex as compared to age-matched TD participants (Foxe et al., 2016). For example, AEP from an individual with Rett syndrome, see Figure 1.

**Table 1:** Summary of evoked potential (EP) and quantitative electroencephalography (EEG) studies of Rett syndrome and related developmental encephalopathies (DEs).

Study	*n*	Age Range	Stimuli	Main Findings
Auditory				
Stach et al. (1994)	36	2–28 years	Clicks, Tones	Normal ABR in all; Abnormal middle and late AEPs in an increasing percentage of patients
Foxe et al. (2016)	14	3–21 years	Tones (oddball)	AEP is abnormal; MMN is present but abnormal
Peters et al. (2015)	Rett: 5 MDD: 12	3–11 years	Familiar and unfamiliar voices	Greater gamma for familiar voice in MDD; Greater gamma for unfamiliar voice in Rett
Peters et al. (2017)	Rett: 9 MDD: 7	4–12 years	Familiar and unfamiliar names	Larger ERPs to own name in MDD; Larger ERPs to another name in Rett
Key et al. (2019)	11	4–12 years	Words and non-words	More negative ERP amplitude to words than non-words at right temporal sites (compared to left temporal sites in TD)
***Visual***
Saunders et al. (1995)	11	4–24 years	Grating stimuli; Reversing checkerboard	Normal visual thresholds; Decreased VEP amplitude and varying latencies
LeBlanc et al. (2015)	34	22 months–8 years	Reversing checkboard	Decreased VEP amplitude; Reduced visual-spatial acuity
Boggio et al. (2016)	FOXG1: 3	17 months–22 years	Light flashes	VEPs in the normal range
***Somatosensory***
Yoshikawa et al. (1991)	10	3–19 years	Median nerve stimulation	SEPs abnormal in 7; Giant SEPs in 5
Guerrini et al. (1998)	10	3–20 years	Median nerve stimulation	SEPs delayed and enlarged
***Multisensory***
Verma et al. (1987)	9	2–15 years	Light flashes; Clicks; Median nerve stimulation	Normal evoked potentials in all participants
Bader et al. (1987)	6	10–22 years	Clicks; Median nerve stimulation	Abnormal SEPs in all participants; Abnormal ABR in all but one
Bader et al. (1989b)	9	10–22 years	Light flashes; Clicks	Slow and distorted VEPs; Early AEP components intact, later components delayed but variable
Kálmánchey (1990)	5	18 months–4 years	Light flashes; Clicks; Median nerve stimulation	Normally evoked potentials in all participants
Yamanouchi et al., 1993	9	2–19 years	Light flashes; Median nerve stimulation	Mechanisms of giant VEPs and SEPs in Rett differ from those of giant EPs in photosensitive progressive myoclonus epilepsy
Stauder et al. (2006)	17	2–60 years	Visual patterns; Tones (both oddball)	Prolonged and attenuated ERPs; A decline in ERP amplitude with increasing age
***Quantitative EEG***
Khwaja et al. (2014)	10	2–10 years	n/a	Decreased right frontal alpha asymmetry between pre- and post-treatment with IGF-1
Ammanuel et al. (2015)	10	2–9 years	n/a	Abnormal delta power during slow-wave sleep
Fabio et al. (2016)	34	5–36 years	n/a	Changes in beta and theta power following cognitive intervention
Keogh et al. (2018)	42	1–23 years	n/a	Differential patterns of interelectrode coherence in individuals with MECP2 vs. CDKL5 mutations
Roche et al., 2019	57	23 months–10 years	n/a	Increased power in lower frequency bands, decreased power in middle-frequency bands

*n—number of participants in the clinical group (Rett syndrome unless otherwise noted), MDD—MECP2 duplication disorder*.

In addition to examining basic AEPs, several studies attempted to examine higher-order auditory processing in Rett syndrome using the so-called “oddball” paradigm. An oddball paradigm presents an infrequent (deviant) tone randomly amongst a string of more frequent (standard) tones. In TD children and adults, the presentation of the deviant stimulus elicits an enhanced amplitude “mismatch” response in the ERP, which is presumed to reflect the detection of a change in stimulus parameters. The existing studies that have used an auditory oddball paradigm with individuals with Rett syndrome have suggested that these mismatch responses are retained in this population, yet attenuated compared to those of controls, reflecting deficits in the underlying cortical networks (Bader et al., 1989b; Stauder et al., 2006; Foxe et al., 2016). The first two studies to utilize this approach were limited by small sample sizes and statistical power but provided initial evidence for the discrimination between frequent and infrequent tones in individuals with Rett syndrome (Bader et al., 1989b; Stauder et al., 2006). Foxe et al. (2016) provided more direct evidence for auditory mismatch responses in individuals with Rett syndrome as part of their study on auditory processing in 14 girls with confirmed MECP2 mutations. Compared to age-matched TD girls, girls with Rett syndrome exhibited a delayed and prolonged mismatch response, which was interpreted as reflecting a slowing of information processing in the Rett group.

Another line of research on auditory processes in Rett syndrome has focused on electrophysiological responses to speech stimuli. While still a new area of research, existing studies of this type have suggested that EPs to speech stimuli may be useful for indexing higher-order language and cognitive processes in individuals with Rett syndrome and related DEs. The first study in this area examined changes in gamma band power in response to familiar and novel voices in children with Rett syndrome and MECP2 duplication syndrome (Peters et al., 2015). While both groups demonstrated electrophysiological evidence of discriminating between the familiar and novel voice, the relative changes in gamma power were in opposite directions, suggesting that over- vs. under-expression of the MECP2 protein has differential effects on the underlying cortical processes. In a second study, Peters et al. similarly noted differences in the electrophysiological responses of children with Rett syndrome and MECP2 duplication syndrome, in this case, to own name vs. other names (Peters et al., 2017). Children with MECP2 duplication syndrome exhibited more positive EPs for own vs. novel names and the extent of this effect was associated with a behavioral measure of adaptive functioning. No significant name discrimination effects were noted for participants with Rett syndrome. More recently, Key et al. (2019) reported more negative EPs to words vs. non-words in girls with Rett syndrome, although this effect was observed in the opposite hemisphere compared to TD controls. Within the Rett group, more typical responses were associated (at trend level) with higher scores on a behavioral measure of receptive vocabulary.

### Visual Evoked Potentials

Visual evoked potentials (VEPs) can be elicited using either patterned or unpatterned “flash” visual stimuli. Initial studies of visual processes in Rett syndrome focused on flash VEPs, with inconsistent results. Whereas two studies reported normal VEPs (Verma et al., 1987; Kálmánchey, 1990), another study presented a distorted waveform and significantly delayed P1 component in participants with Rett syndrome (Bader et al., 1989b). Differences in age may have contributed to the disparate results across these studies.

Subsequent studies on VEPs in Rett syndrome have typically measured responses to patterned visual stimuli, which have less intra- and inter-subject variability and greater sensitivity than those for flash stimuli (see Table 1 for a summary of studies). The earliest patterned stimuli study reported that the VEP waveforms of 10 girls with Rett syndrome appeared subjectively different than those of TD children, particularly in regards to P1 amplitude and N2 latency (Saunders et al., 1995). However, these differences did not reach statistical significance, which the authors attributed to the small sample size and high level of individual variability in both the Rett and control groups.

A recent study with a relatively large sample of 34 girls with Rett syndrome detected significant differences in several aspects of the VEP of individuals with Rett syndrome compared to TD controls (LeBlanc et al., 2015), which paralleled the findings of Saunders et al. (1995). The most striking difference was attenuation of the P1 component in individuals with Rett syndrome as indexed by both N1—P1 and P1—N2 interpeak amplitudes. The Rett group also showed delays in N2 latency, as measured by absolute peak latency as well as P1—N2 time. Further analyses revealed that these effects were particularly prominent in the later stages of the disorder. Specifically, when the larger group of 34 participants was subdivided into either active- or post-regression, the most notable differences in N1—P1 amplitude and P1—N2 time was for the post-regression vs. TD groups, with participants in active-regression falling in the middle. In addition to examining basic VEPs, LeBlanc et al. (2015) also recorded VEPs to varying spatial frequency in a smaller number of participants to evaluate visual acuity in this population. The pattern of findings indicated reduced spatial frequency sensitivity and diminished acuity in the Rett group, with a dominant spatial frequency of 0.4 cpd vs. 1.4 cpd for controls. Of note, the primary findings of diminished visual acuity and a decline in VEP amplitude with disease progression were also found in a parallel study with MECP2 deficient mice (see “Research with Animal Models” section). In addition to providing further support for the reliability of the human findings, the comparable results in mice point to the potential utility of VEPs as a biomarker, which, in an ideal case would be translatable between species (see “Discussion” section).

Only one electrophysiological study to date has considered higher-order visual processing in Rett syndrome. This study by Stauder et al. (2006) utilized an oddball design to examine how individuals with Rett syndrome process novel visual information. Compared to TD controls who demonstrated larger responses to novel vs. frequent visual stimuli, the responses for the individuals with Rett syndrome did not clearly discriminate between the two trial types. This was particularly true for older participants (15–60 years of age), who failed to show any difference for novel vs. frequent stimuli, leading the authors to conclude that individuals with Rett syndrome show a marked decline in ERP task modulation with increasing age.

Overall, the existing studies on VEPs in Rett syndrome suggest that similar to AEPs, these responses are atypical in this population, particularly when elicited using patterned visual stimuli (see Figure 1 for a VEP from an individual with Rett syndrome). This work has further pointed to the potential influence of the clinical-stage on the VEP waveform with more atypical responses in later stages of the disorder. However, the existing studies on VEPs in Rett syndromes have been limited by small sample sizes (Verma et al., 1987; Bader et al., 1989b; Kálmánchey, 1990; Saunders et al., 1995; Stauder et al., 2006) or relatively restricted age ranges (LeBlanc et al., 2015). Therefore, additional work with larger samples and wider age ranges are needed to fully decipher the association between VEP parameters and disease progression.

Currently, it is not known how VEPs are affected in the related DEs (MECP2 duplication syndrome, CDD, or FOXG1 disorder). Preclinical studies with CDKL5- and FOXG1-mutated mice indicate that these responses are atypical in animal models of these conditions (see “Research With Animal Models” section). However, one paper reporting abnormal VEPs in FOXG1 mutated mice failed to find similar abnormalities in three human participants for whom VEPs were acquired but used different stimuli between the mice (contrast reversal) and the FOXG1 subjects (Strobe flash) tested (Boggio et al., 2016). Further work with larger samples of individuals with FOXG1 and the other syndromes will be needed to fully delineate characteristics of the VEP in these populations.

### Somatosensory Evoked Potentials

Relatively less attention has been given to somatosensory processes in Rett syndrome as compared to auditory and visual processes, particularly in recent years. Overall, research in this area has indicated delayed responses and prolonged conduction times in individuals with Rett syndrome compared to normative comparison groups. Studies have reported normal latencies for the initial component following electrical stimulation of the median nerve (N9) over Erb’s point in all participants, but delays in the subsequent cervical N13 and cortical N20 components in more than half of the individuals tested. In addition to delays in absolute latencies of these components, prolonged N13—N20 and N20—P30 interpeak intervals have also often been observed in a majority of individuals, further suggesting a slowing in central somatosensory pathways in Rett syndrome (Bader et al., 1987, 1989a; Kimura et al., 1992; Guerrini et al., 1998). Kimura et al. (1992) noted that these delays were most apparent in children over 9 years of age, with normal SEPs for younger children, pointing to a potential degenerative process with increasing disease duration.

In addition to noting differences in SEP latency, studies on somatosensory responses in Rett syndrome have also noted enhanced or “giant” cortical SEPs in a subset of participants (Yoshikawa et al., 1991; Yamanouchi et al., 1993; Guerrini et al., 1998). Giant SEPs are also observed with high incidence in individuals with cortical myoclonus and are presumed to reflect altered excitability within the somatosensory cortex. To better understand the pathophysiology of the enhanced SEPs in Rett syndrome, Yamanouchi et al. (1993) directly compared SEPs in nine girls with Rett syndrome with six children with progressive myoclonus epilepsy. Giant SEPs, defined as more than 3 standard deviations of the mean for age-matched controls, were observed in all of the individuals with progressive myoclonus epilepsy, but only six individuals with Rett syndrome. Another study reported giant SEPs in a similar proportion of Rett participants (Yoshikawa et al., 1991). These authors noted the individuals with giant SEPs tended to be younger (<9 years of age) and speculated that giant SEPs may be specific to earlier stages of the disorder and decline in later stages when seizures are less common. Further work with a larger sample is needed to confirm this suggestion and the associations between giant SEPs and epilepsy among individuals with Rett syndrome.

Although most studies on SEPs in Rett syndrome have reported atypical responses as compared to TD groups, two studies found no differences in the SEPs of Rett vs. control participants (Verma et al., 1987; Kálmánchey, 1990). Of note, these studies were also among the few that reported normal AEPs and VEPs in Rett syndrome. The participants were relatively young (mostly under 10 years of age) compared to the wider age ranges used in other studies. As described above, Kimura et al. (1992) specifically noted that SEPs were normal in children under 9 years of age. Together, with the findings from VEPs, these findings point to a potential decline in EPs with disease progression in Rett syndrome and reinforce the need for future work to explicitly examine how EPs change throughout the disorder. To our knowledge, no studies have been done on SEPs in MECP2 duplication syndrome, CDD, or FOXG1 disorder.

### EEG Analysis

Abnormal background EEG has been considered a common feature of Rett syndrome since its initial characterization (Rett, 1966; Hagberg et al., 1983). Several articles have since described these abnormalities in detail (Niedermeyer et al., 1986; Verma et al., 1986; Glaze et al., 1987; Garofalo et al., 1988; Hagne et al., 1989; Ishizaki et al., 1989). A thorough review of this literature is beyond the scope of this article, but generally, this work has demonstrated that the most common abnormalities are diffuse slowing of the background EEG and the presence of epileptiform activity, even in individuals without a history of seizures (Niedermeyer et al., 1986; Garofalo et al., 1988; Glaze, 2002, 2005). These abnormalities tend to follow a characteristic developmental course with a pattern of largely normal EEG before regression followed by the onset of spike and sharp waves that are initially most prominent over centrotemporal regions and then become more generalized in distribution (see Glaze, 2002, 2005). These epileptiform abnormalities tend to decline in the late stages of the disorder, although the slowing of the background EEG is still apparent at this stage, particularly in the theta band over frontal-central regions.

While this literature has substantially advanced the understanding of electrophysiological abnormalities in Rett syndrome, the inferences were based primarily on visual inspection of the data. The use of resting EEG as an efficacy biomarker for clinical trials will likely require a more reproducible, quantitative approach. Few studies on resting EEG in Rett syndrome have applied quantitative EEG analysis, although these methods have been used extensively to study TD and other neurodevelopmental disorders (Saby and Marshall, 2012; Wang et al., 2013; Bick and Nelson, 2016; Heunis et al., 2016). Common approaches to quantitative analysis of resting EEG include spectral power analysis, in which the EEG signal is decomposed into component frequency bands (delta, theta, alpha, beta, and gamma) and coherence, which estimates the degree to which two areas of the brain are “networked” together by determining the similarity in neuronal oscillations between electrodes or regions.

The few studies that have applied quantitative analyses to resting EEG in Rett syndrome have provided some indication that spectral power measures are sensitive to treatment, and thus may represent a valuable objective biomarker for clinical trials. As part of the Phase-1 clinical trial on mecasermin (IGF-1) in Rett syndrome, Khwaja et al. (2014) reported a reduction in right frontal alpha asymmetry between the pre- and post-treatment period. Right frontal alpha asymmetry, which indicates greater alpha power at right vs. left frontal electrodes, has been associated with increased internalizing behaviors, including anxiety and depression (Thibodeau et al., 2006). Thus, the finding of a reduction in right frontal alpha asymmetry was considered to index a decrease in anxiety symptoms following IGF-1 treatment. This conclusion was supported by a trend-level reduction in anxiety on a standardized behavioral assessment of anxiety symptoms. Subsequently, Fabio et al. (2016) reported increased beta and decreased theta power in the resting EEG of girls with Rett syndrome following five days of cognitive training, suggesting that spectral power measures may be sensitive to even brief interventions.

Keogh et al. (2018) demonstrated that inter-electrode coherence may also prove useful as an EEG biomarker for Rett syndrome and related DEs. In this study, spectral power and inter-electrode coherence measures were calculated from the resting EEG of individuals with MECP2 and CDKL5 mutations. The results indicated no differences in spectral power between the two groups, but differing patterns of inter-electrode coherence, particularly in occipital and temporal regions. Furthermore, different patterns of inter-electrode coherence were also observed for different subgroups of individuals with MECP2 mutations, namely those with Classic Rett vs. Preserved Speech Variant, and for subgroups of individuals with epilepsy (absent, present, or treatment-resistant). No significant differences in spectral power were observed for the MECP2 vs. CDKL5 or subgroups comparisons, suggesting that inter-electrode coherence may be more specific to individual groups than power measures. Recently, Roche et al. (2019) performed EEG on 57 Rett syndrome subjects and 37 age-matched controls to conclude that EEG frequency spectral composition partially correlated with lower cognitive assessment scores. EEG power was measured and compared to controls and between active regression and post regression states with general finding of slower (higher power in the delta and theta frequencies) EEG which reached statistical significance in particular head regions (Roche et al., 2019). Finally, these authors reported that the higher log-transformed delta power was associated with lower developmental quotients.

In addition to studies of background EEG during wakefulness, there has also been an interest in EEG patterns during sleep among girls with Rett syndrome. Building on descriptive studies of EEG abnormalities during sleep in Rett syndrome, Ammanuel et al. (2015) applied quantitative EEG analyses to further characterize differences in the sleeping EEGs of girls with Rett syndrome and age-matched controls. The primary finding was that participants with Rett syndrome exhibited greater delta power during slow-wave sleep and that delta power in the Rett group did not decline overnight as it did in control participants. While these findings suggest that delta power may be useful as a biomarker for sleep dysfunction in Rett syndrome, this study lacked the power to determine how these measures related to sleep quality at the individual level.

## Research With Animal Models

The clinical EP and EEG studies described above, while not exhaustive, present evidence of visual, auditory, somatosensory, and resting EEG changes in Rett subjects vs. TD controls that could be used as prognostic or predictive biomarkers in the DEs. The animal literature generally supports the existing human studies, but with expected differences. As with the human studies, there are many papers on different Rett, *MECP2* duplication, *FOXG1*, and *CDKL5* mouse models, many of which study the behavioral, anatomic, molecular, and physiological changes that loss or mutation in these genes cause (Guy et al., 2001; Collins et al., 2004; Wang et al., 2012; Boggio et al., 2016). And further in line with the human studies, there are fewer animal model EP/EEG studies then there is work on the cellular and molecular biology of Rett syndrome. In agreement with the human studies, most of the research within the DEs have been done on Rett syndrome (*Mecp2* mutant) mice but with a scattering of studies on the other disorders. In all cases, much of the research has been done with EEG, followed by evoked potential studies.

There are a host of Mecp2 deficient mouse line studies and reviewing the individual findings on them is beyond the scope of this review (for review see Vashi and Justice, 2019). In almost all lines, there has been a consistent EEG finding of 5–9 Hz sharps in runs lasting 1–2 s (D’Cruz et al., 2010; Eubanks, 2017; Wither et al., 2018). These discharges have been demonstrated to decrease in frequency and content with certain drugs, mainly those that treat absence seizures in humans: valproic acid and ethosuximide (Wither et al., 2018). A few authors have shown that these discharges change with the severity of the disease in the mouse (for review see Eubanks, 2017). Detailed quantitative analysis of the EEG, by frequency measures (D’Cruz et al., 2010; McLeod et al., 2013; Colic et al., 2014) or network measures (Colic et al., 2015) have also demonstrated changes with age/severity and some which could predict response to drugs (Wither et al., 2018). The EEG findings in the *Cdkl5* mice have been normal, both in quality and quantifying frequency content (Wang et al., 2012). The Mecp2 duplication mouse line has intermittent epileptiform discharges on EEG (Collins et al., 2004). As a whole, these studies have demonstrated that the EEG mimics findings in humans and is a potential biomarker for disease course and outcome for preclinical trials.

Evoked potentials studies in Mecp2 mutant mice first showed no differences in the brain stem component of the AEP (Liao et al., 2012). These studies did show differences in the middle latency auditory and visual cortical components (Liao et al., 2012) in an exon 4 deletion mouse and subsequent studies in a missense mutation mouse showed similar findings (Goffin and Zhou, 2012). These authors also demonstrated differences in frequency coupling to the stimuli suggesting local circuit dysfunction but in different directions for the two lines (Goffin and Zhou, 2012; Liao et al., 2012). Similar increases were reported in the phase-locking factor that suggests a hyper-synchronous response to stimuli (Goffin and Zhou, 2012; Liao et al., 2012). Follow up studies by this group, demonstrated that these findings could be rescued by restoration of Mecp2 in gabaergic neurons (Goffin et al., 2014). More recent visual evoked potential studies have confirmed that there is a difference in amplitude of the VEP in *Mecp2* mice that track with disease severity and closely mirrors the human findings (see above, LeBlanc et al., 2015). A Mecp2 rat study has demonstrated that the Mecp2 mutant rats had hyperexcitable but slower responses to speech sounds across the auditory cortex. They found that the Mecp2 rats could perform consonant and vowel discrimination tasks, but this ability was impaired when the stimuli were presented with background noise. Extensive speech training improved the Mecp2 rat’s performance, but differently than control rats (Engineer et al., 2015).

Studies of the AEP in *Cdkl5* mice have also demonstrated a reduction in the amplitude of the N1 and P2 responses with a change in latency of the P2 response as well as a shift in the phase-locking factor (Wang et al., 2012). Two studies by the Pizzorusso et al., first using optical blood flow imaging (Mazziotti et al., 2017; Lupori et al., 2019), then repeated with cortical EPs (Mazziotti et al., 2017), demonstrated no differences in cortical optical imaging responses at the first age tested (P25–P26), but these emerged days later (P27–P28) in *Cdkl5* mutant mice. A second study demonstrated a reduced VEP response in the mutant Cdkl5 mice at both age P28 as well as in more mature animals (P80). Other work has shown that in the Foxg1 deletion mouse line there is a reduction in visual acuity and response amplitude in visual cortex recordings in response to different visual stimuli (Boggio et al., 2016).

Together, the preclinical rodent models of the DEs present evidence for altered physiological responses that suggest both short and long-range cortical dysfunction. These findings could be used for biomarker studies for preclinical drug development along with the human EP studies described above.

## Discussion

Overall, this review demonstrates that EP and EEG measures are abnormal in individuals with Rett syndrome. Although limited, extant studies that have included participants with related DEs (MECP2 duplication syndrome and CDD) suggest that EPs and EEG measures are also affected in these disorders, albeit in a distinct fashion (Peters et al., 2015, 2017; Keogh et al., 2018). Studies at the preclinical level have similarly noted striking abnormalities in EP and EEG measures in animal models of these conditions (e.g., Goffin et al., 2014; Boggio et al., 2016; Mazziotti et al., 2017). The finding that electrophysiological measures are atypical in Rett syndrome and the related DEs, as well as in animal models of these disorders, suggests that these measures may have future utility as an objective marker of disease progression or treatment response. In a clinical trial, a shift in the EP/EEG waveform could indicate a response to treatment. However, several important questions must be addressed before we can translate these measures into biomarkers for clinical use.

The existing literature has identified many EP and EEG measures that appear to be affected in individuals with Rett syndrome. In the auditory domain alone, the latency and amplitude of the cortical components of the AEP response to basic tones (Bader et al., 1989b; Stach et al., 1994) as well as measures of higher-order auditory processes such as the mismatch negativity (Foxe et al., 2016) and evoked responses to speech sounds (Peters et al., 2017; Key et al., 2019) are abnormal in individual groups. Other work has shown that aspects of the VEP (LeBlanc et al., 2015), SEP (Bader et al., 1989a; Kimura et al., 1992; Guerrini et al., 1998), and resting EEG (Keogh et al., 2018; Roche et al., 2019) are also atypical. One pressing question for future work concerns which of these electrophysiological measures are the most robust and valid indicators of function and thus, represent good candidate biomarkers to pursue for qualification.

A substantial limitation of the existing work on EP/EEG measures in Rett syndrome is small sample sizes. With few exceptions (e.g., LeBlanc et al., 2015; Roche et al., 2019), existing studies in this area have typically enrolled between 5 and 15 individuals. Future research with larger samples is needed to confirm the findings from these smaller studies and importantly, elucidate how these measures relate to function. As described above, many of the extant studies noted considerable variability in the responses within the individual group, ranging from apparently normal to, in the case of EPs, completely absent responses. However, due to small Ns, most of these studies did not attempt to address the potential clinical significance of this variability. The few studies that did include brain-behavior correlations largely failed to find significant associations, likely owing to the use of small samples (Peters et al., 2015, 2017; Key et al., 2019). To precisely identify how EP and EEG measures relate to function in Rett syndrome, a study with a sufficiently large sample is needed. To fully decipher these brain-behavior associations, this sample must be not only large but also representative of the heterogeneous population of girls and women with Rett syndrome, encompassing individuals of all ages and with differing degrees of clinical severity.

In addition to a large study on Rett syndrome, more research into the related DEs (MECP2 duplication syndrome, CDD, and FOXG1 disorder) is needed. Very few EP and EEG studies have included participants with these conditions. Those that have reported different electrophysiological patterns among participants with MECP2 duplication syndrome (Peters et al., 2015, 2017) and CDD (Keogh et al., 2018) as compared to participants with Rett syndrome. Therefore, biomarkers of function for these disorders will have to be validated separately from those for Rett syndrome. Due to the low incidence of these conditions, research on these conditions type will undoubtedly require data collection at multiple sites. The most informative approach would involve applying the same methods in participants with Rett syndrome, MECP2 duplication syndrome, CDD, FOXG1 disorder, and TD controls to directly assess how EP and EEG measures in these disorders vary compared to TD and one another. An analogous study with animal models of Rett syndrome and each of the related DEs would also be extremely valuable for advancing the understanding of the similarities and differences in EP/EEG measures across these disorders.

In addressing the question of which EP/EEG measures reliably reflect function in Rett syndrome and related DEs, it is important to consider that different trials will likely require different biomarkers, depending on the nature of the treatment under study. Many of the therapeutics under development for the DEs aim to improve global functioning and reduce symptoms across a variety of domains. Others target a particular symptom such as seizures or breathing abnormalities. For trials evaluating therapeutics to improve function more generally, electrophysiological measures that are sensitive to overall neurologic functioning will be the most fitting. For trials evaluating therapeutics with more specific targets, electrophysiological measures that more specifically correlate with the severity of the symptom of interest will be more appropriate.

In addition to being sensitive to function, an ideal biomarker would also be translatable. Currently, there is a substantial divide in the outcome measures used in preclinical studies with animal models and those used in clinical trials with patient groups. Specifically, at the preclinical level, efficacy is typically assessed using animal-specific behaviors and changes at the cellular level such as increasing dendritic spine density or long-term potentiation. Considering efficacy in humans is based on caregiver or clinician impression of observable changes in function, it is not surprising that many treatments with proven efficacy in mice have failed to show similar effects in humans. If preclinical results are expected to persist in clinical trials, a more fruitful approach would be to use the same measures in animals that we do in humans. Many of the candidate EP and EEG measures described in this review are likely to be translatable in this way. For instance, LeBlanc et al. (2015) demonstrated that VEPs elicited and analyzed from mice and humans using parallel methods yield comparable results. Future studies should continue to apply the same methods with mice and humans to identify which candidate electrophysiological biomarkers are most translatable. Recently, a primate model of Rett syndrome has been generated using Talon DNA editing technology (Liu et al., 2016; Chen et al., 2017). These models recapitulate some of the features of Rett syndrome and could be excellent in-between steps from mouse to humans to test the validity of these potential biomarkers. Unfortunately, primate studies are often expensive and have limitations that could make going straight from rodent to human more feasible. Since many compounds have already proven effective at the preclinical level, future work with animal models is also needed for identifying which candidate EP and EEG measures may be the most responsive to treatment, an additional requirement for these measures to be useful as biomarkers in clinical trials.

Once candidate biomarkers are identified, it will also be necessary to understand their development. The issue of age-related changes in biomarkers is a particular challenge for biomarker discovery for neurodevelopmental disorders since most biological measures, including EPs and EEG measures, are known to change with development (McPartland, 2016; Sahin et al., 2018). It is, therefore, necessary to understand how candidate biomarkers change in the absence of treatment to more appropriately gauge improvement in the presence of an intervention. Indeed, several studies have indicated that EP measures may decline with age or disorder progression in individuals with Rett syndrome (Kimura et al., 1992; Stauder et al., 2006; LeBlanc et al., 2015). Studies will need to consider this decline when examining the effect of treatment over a long period. Furthermore, this raises the question of whether the same EP and EEG biomarkers will be valid across all ages or whether different biomarkers will be needed for individuals of different ages or in different stages of the disorder. Large studies with participants of different ages are needed to help decipher developmental changes in these measures and the degrees to which they reliably reflect neurological functioning.

Addressing these questions and validating EP/EEG biomarkers for clinical trials of Rett syndrome and related DEs will not be without challenges. Although EEG is a relatively fitting neuroimaging technique for use with individuals with profound disabilities, obtaining good-quality data from this population is often difficult and EEG artifacts arising from behavioral movement, teeth grinding, and breathing abnormalities are common (Gabard-Durnam et al., 2018). Paradigms also have to be relatively short in duration and, therefore the number of trials is often less than optimal. Another significant challenge of this work relates to the low incidence of Rett syndrome (~1 in 10,000 females) and particularly, the related DEs, which are estimated to occur in less than 1 in 100,000. For this reason, qualifying biomarkers of these disorders will require multi-site collaborations and potentially the use of large control data sets to achieve sufficiently powered samples. While multi-site research is undoubtedly beneficial for increasing power and generalizability, it also introduces a range of methodological challenges, including the need to rigorously standardize stimulus presentation and data acquisition methods. Lastly, although EPs and EEG measures have been studied in Rett syndrome and appear to have potential utility as biomarkers for efficacy endpoints in clinical trials, it should be noted that other types of biomarkers may also be useful for this purpose. This includes brain-based measures, including magnetoencephalography or transcranial magnetic stimulation, pupillometry, and sympathetic testing, as well as physiological/behavioral measures derived from wearable sensors. Although few studies to date have utilized these methods in participants with Rett syndrome (Heinen and Korinthenberg, 1996; Heinen et al., 1997; Krajnc and Zidar, 2016; Santosh et al., 2017; Artoni et al., 2019), these approaches have proven useful in biomarker research for other neurodevelopmental disorders (Roberts et al., 2010; Oberman et al., 2016; Ness et al., 2017).

## Conclusion

Rett syndrome, MECP2 duplication syndrome, CDD, and FOXG1 disorder are severe neurodevelopmental conditions that result in life-long impairment across multiple domains of functioning. Treatment options for these disorders are currently very limited. However, promising therapeutics are now being investigated in animal models, with many of these treatments likely to proceed to clinical trials in the coming years. The success of these trials is likely to benefit from the identification of biological markers to objectively quantify neurological function in individuals with Rett syndrome and the related DEs, thus reducing the reliance on caregiver/clinician impression scales which are inherently subjective and subject to placebo effects. Various electrophysiological measures (EPs and resting EEG) are abnormal in individuals with Rett syndrome and representative animal models and thus, embody candidate biomarkers to monitor response to treatment. However, before we can apply these measures as endpoints in clinical trials, several important questions related to the functional significance, development, and progression of these biomarkers need to be addressed. Given Rett syndrome and particularly the related DEs are rare conditions, these questions will be best resolved by multi-site studies to achieve more robust and representative samples.

## Author Contributions

JS conceived of, wrote and edited the manuscript. EM conceived of, wrote, and was editor of the manuscript. TR, CN and SP conceived of and edited the manuscript.

## Conflict of Interest

Dr. TR declared positions on medical/scientific advisory boards or service as a consultant for CTF, Ricoh, Prism Clinical Imaging, Spago Nanomedicine, Avexis and Acadia Pharmaceuticals. He also declares intellectual property concerning MEG as a biomarker for clinical trials in ASD. The remaining authors declare that the research was conducted in the absence of any commercial or financial relationships that could be construed as a potential conflict of interest.
